# Clinical presentation, genotypic diversity, and intracellular bacteria in *Acanthamoeba* keratitis patients treated at a referral eye hospital in Sydney, Australia

**DOI:** 10.1016/j.ijregi.2025.100690

**Published:** 2025-06-15

**Authors:** Binod Rayamajhee, Mark Willcox, Fiona L. Henriquez, Gauri Sr Shrestha, Uday Narayan Yadav, Amberin Fazal, Sheng Chiong Hong, Alexander Chorny, Yalewayker Asrat, Constantinos Petsoglou, Nicole Carnt

**Affiliations:** a School of Optometry and Vision Science, Faculty of Medicine and Health, UNSW, Sydney, NSW, Australia; b School of Biological, Earth and Environmental Sciences (BEES), UNSW, Sydney, NSW, Australia; c Department of Infection and Immunology, Kathmandu Research Institute for Biological Sciences (KRIBS), Lalitpur 44700, Nepal; d Department of Civil and Environmental Engineering, University of Strathclyde, Glasgow, G1 1XJ, Scotland, UK; e Department of Ophthalmology, Institute of Medicine, Kathmandu, Nepal; f National Centre for Epidemiology and Population Health, The Australian National University, Canberra, ACT, Australia; g International Centre for Future Health Systems, UNSW, Sydney, NSW, Australia; h Sydney and Sydney Eye Hospital, Southeastern Sydney Local Health District, Sydney, NSW, Australia; i Save Sight Institute, University of Sydney, Sydney, Australia

**Keywords:** *Acanthamoeba* keratitis, Contact lens, Genotypes, Epidemiology

## Abstract

**Objectives:**

*Acanthamoeba* keratitis (AK) is an emerging corneal infection. This study evaluated the prevalence of AK, assessed circulating *Acanthamoeba* genotypes, and identified potential sources of infection.

**Methods:**

A prospective case series study was conducted at the Sydney Eye Hospital, Australia, from June 2021 and October 2022. Corneal swabs from AK patients were collected for culture, and their domestic water samples were also analyzed. Cyst morphology, polymerase chain reaction, and Sanger sequencing were performed to confirm the *Acanthamoeba* isolates.

**Results:**

A total of 21 AK patients were recruited in this study (41 ± 12.3 years). Six (28.6%) corneal and four (44.4%) water samples tested positive for *Acanthamoeba* genotype T4. One corneal and one water isolate harbored intracellular bacteria, with the water isolate containing *Pseudomonas aeruginosa*. Eight patients were contact lens wearers. Eye pain was the primary symptom (66.7%), followed by red eye. The median duration of symptoms was 24.5 days (interquartile range: 13–95 days). Polyhexamethylene biguanide was the main therapy, followed by chlorhexidine. The median treatment duration was 12 weeks (interquartile range: 8–26 weeks).

**Conclusions:**

In Sydney, the prevalent genotype of *Acanthamoeba* among AK patients and tap water was T4. This study suggests a higher annual incidence of AK than previously reported.

## Highlights

• *Acanthamoeba* keratitis is an emerging sight-threatening corneal infection.
• This study observed a relatively high incidence of *Acanthamoeba* keratitis in Sydney.
• Genotype T4 is the most prevalent *Acanthamoeba* associated with keratitis in Australia.
• Intracellular bacteria in *Acanthamoeba* can contribute to polymicrobial keratitis.

## Graphical abstract

**Figure:**
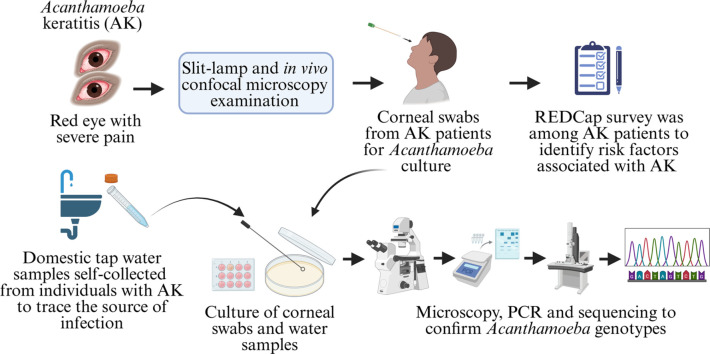

## Introduction

*Acanthamoeba* species are widespread free–living amoebae found in soil and water [1]. They are opportunistic pathogens of humans. *Acanthamoeba* has a biphasic life cycle, comprising a large, metabolically active trophozoite (12–35 μm) and a smaller, inactive cyst (5–20 μm) [2]. Given its widespread presence in the environment, humans are susceptible to exposure and healthy individuals may have anti–*Acanthamoeba* antibodies [3]. *Acanthamoeba* was first reported as the causative agent of keratitis (AK) both in the UK and the USA in 1973 [4,5]. Subsequently, there has been a rising incidence of AK, which has been linked to the increased use of contact lenses and inadequate hygiene when wearing lenses [6]. The clinical presentation of AK can closely mimic that of other forms of keratitis, such as Herpes simplex keratitis (HSV) [7], contributing to its frequent misdiagnosis. Culture of corneal specimens remains the gold standard for diagnosing AK; however, culturing *Acanthamoeba* can be challenging, and the sensitivity varies, with rates ranging from 7% [8] to 74% [9] or 82% [10]. Significantly delayed diagnosis can result from the prolonged incubation required for *Acanthamoeba* culture [11]. Enhancing both the specificity and sensitivity of AK diagnosis is vital for ensuring a successful treatment outcome [9,12,13].

An estimated 10–40 cases of AK occur annually in Australia, with higher reported incidences in Brisbane (1.7 per million) [14] and Melbourne (1.3 per million) [15] compared to New South Wales (0.5 per million) [16]. A retrospective study of 52 AK patients at Sydney Eye Hospital (2002–2016) found 8% had bacterial or viral coinfections, 83% were contact lens wearers, and 17.6% developed AK after swimming in fresh or seawater while wearing lenses [16]. This could potentially be attributed to the coastal location of the city or contact with domestic tap water while wearing lenses, as keratitis-causing *Acanthamoeba* species have been reported in public lagoons [17] and domestic tap water within the Sydney metropolitan area [18]. Similar observations were made in Melbourne and Brisbane, where contact lens wear was the primary risk factor for AK (86% and 84%, respectively) [14,15].

The data on AK in Australia are limited, particularly regarding the genotypes among AK cases, with only a few retrospective case series audits in the literature [[14], [15], [16]]. In the present study, corneal swabs obtained from AK patients at a tertiary eye hospital in Sydney were cultured to investigate the epidemiological distribution of *Acanthamoeba rns* genotypes, as well as the associated clinical characteristics of participants. The presence of intracellular bacteria was assessed among culture-confirmed *Acanthamoeba* isolates. Additionally, domestic tap water was assessed to trace the possible source of infection.

## Study site, materials, and methods

A hospital-based prospective case series study was conducted at Sydney Eye Hospital, Sydney, Australia, between June 2021 to October 2022. Patients showing clinical signs of AK underwent a comprehensive slit-lamp examination to investigate the cornea, assess infiltrates, measure ulcer size, determine sclera involvement, identify the presence or absence of foreign particles, and document the status of the ocular adnexa. In addition, *in vivo* confocal microscopy (IVCM) was used to scan for the presence of double-walled amoebal cysts and oval/round hyperreflective spots measuring 8–22 µm in size to aid diagnosis. Ocular swabs with epithelial debridement were then collected using sterile swabs after topically applying anesthetic eye drops.

### Culture and microscopic analysis of Acanthamoeba

The collected corneal swabs, along with Ringer's solution, were directly inoculated onto non-nutrient agar (NNA) plates pre-seeded with heat-killed *Escherichia coli* (ATCC 10798), as described previously [19]. The plates were allowed to absorb the Ringer’s solution for 15–20 minutes at ambient temperature in a class II biological safety cabinet, then parafilm-sealed plates were incubated for up to 4 weeks at 32°C. Following initial isolation, the *Acanthamoeba* strains were cultured on fresh NNA without *E. coli,* then transferred into PYG broth (proteose peptone, yeast extract, and glucose, pH 6.5) supplemented with 250 µl/ml penicillin-streptomycin in a 24-well plate. This study also analyzed domestic tap water samples self-collected from individuals with AK to trace the possible source of infection, as previous studies have found that approximately one-third of bathroom tap water samples in Sydney contain *Acanthamoeba* [20]. A water sample kit was provided to study participants with water collection instructions (Appendix A, supplementary file).

### Polymerase chain reaction amplification and sequencing to confirm Acanthamoeba spp

Isolates showing *Acanthamoeba*-like morphological features were selected for confirmation using polymerase chain reaction (PCR). Genomic DNA was isolated using the DNeasy blood and tissue kit (Qiagen, Hilden, Germany) following the manufacturer’s instructions. The *Rns* gene region of amoebal 18S rRNA was amplified using the *Acanthamoeba* genus-specific primer pair JDP1 and JDP2, as previously described [21]. PCR-positive amplicons were used for Sanger sequencing at the Ramaciotti Centre for Genomics (UNSW, Sydney). Following purification of the PCR products using ExoSAP-IT cleanup reagents, sequencing was performed using BigDye Terminator (V3.1) reaction mixture in a 3730 DNA analyzer. Sequence reads with low quality were trimmed and aligned using the MUSCLE algorithm in MEGA11.

### Assessment of endosymbionts in Acanthamoeba isolates

Axenically grown *Acanthamoeba* trophozoites in PYG broth were assessed for the presence of intracellular bacteria by PCR targeting bacterial 16S rRNA, and fluorescence *in situ* hybridization (FISH) using 16S rRNA probes. This study followed the same protocol as previously outlined [21], except for the PCR primers. In the current study, the primer pair 341Fw and 785Rv was used to amplify 16S V3-4 as described elsewhere [22]. The oligonucleotide probes used in this study for the hybridization assay are listed in Table S1. To confirm the intracellular presence of bacteria in *Acanthamoeba* isolates, transmission electron microscopy (TEM) was performed, following a previously published protocol [23].

### Clinical data of AK patients

Demographic and clinical data of all recruited AK patients were retrieved in a customized Excel datasheet from the health record department at Sydney Eye Hospital by a trained optometrist. The clinical records were assessed for features such as reported symptoms, eye trauma, infection duration, laboratory investigations, size and characteristics of infiltrates and epithelial defects, hypopyon, prior medication, treatment given at the clinic, treatment duration, type of topical medication, treatment outcome, initial and final visual acuity. In addition to clinical data, a REDCap risk factor survey was conducted among AK patients to identify potential risk factors associated with AK (Flow diagram 1, supplementary file).

### Data presentation and analysis

Data analysis was performed in GraphPad Prism V9 (GraphPad Software, CA, USA). Percentage representations were used for proportions, while continuous data were expressed as mean ± standard deviation. A 95% CI was calculated for clinical and demographic data using proportion (p) ± 1.96 × SEM (standard error of mean). An unpaired *t-*test was used to compare clinical and demographic data with the culture-positive *Acanthamoeba* status, using a significance level of *P* < 0.05 for two-tailed tests.

## Results

A total of 21 clinically confirmed AK patients (21 eyes) were recruited in this study, with the highest number of cases observed in January, March, and October 2022 (Figure S1). Twenty patients (95.2%) were from different suburbs of the Sydney metropolitan area, with the remaining one case from the Northern Territory, Australia (Figure S2). Nine patients (42.9%) presented at the study hospital in summer (December-February), followed by eight in spring (38.1%; September-November), with two in each of winter (June-August) and autumn (March-May; Figure S2). Among the 21 patients, nine (42.9%) sent their domestic tap water for *Acanthamoeba* culture, and 12 (57.1%) responded to the REDCap risk factors survey.

### Culture growth and identification of Acanthamoeba spp

Among the 21 corneal swabs collected and cultured, only six (28.6%) grew *Acanthamoeba* (Figure S1), with four (66.7%) of them isolated from specimens collected during the summer months. Out of nine tap water samples collected from the domestic water supply of AK patients, four (44.4%) were *Acanthamoeba* spp. culture positive. Two isolates (50%) were found during the summer, and the remaining two were from the autumn season (Figure S3). All culture-positive isolates were confirmed as *Acanthamoeba* spp. through microscopy followed by PCR and sequencing of the *rns* gene (Figures S4 and S5). *Acanthamoeba-*like cysts were labelled as ‘most probable’ on NNA plates and then sub-cultured onto new NNA to obtain trophozoites.

**Figure 1:**
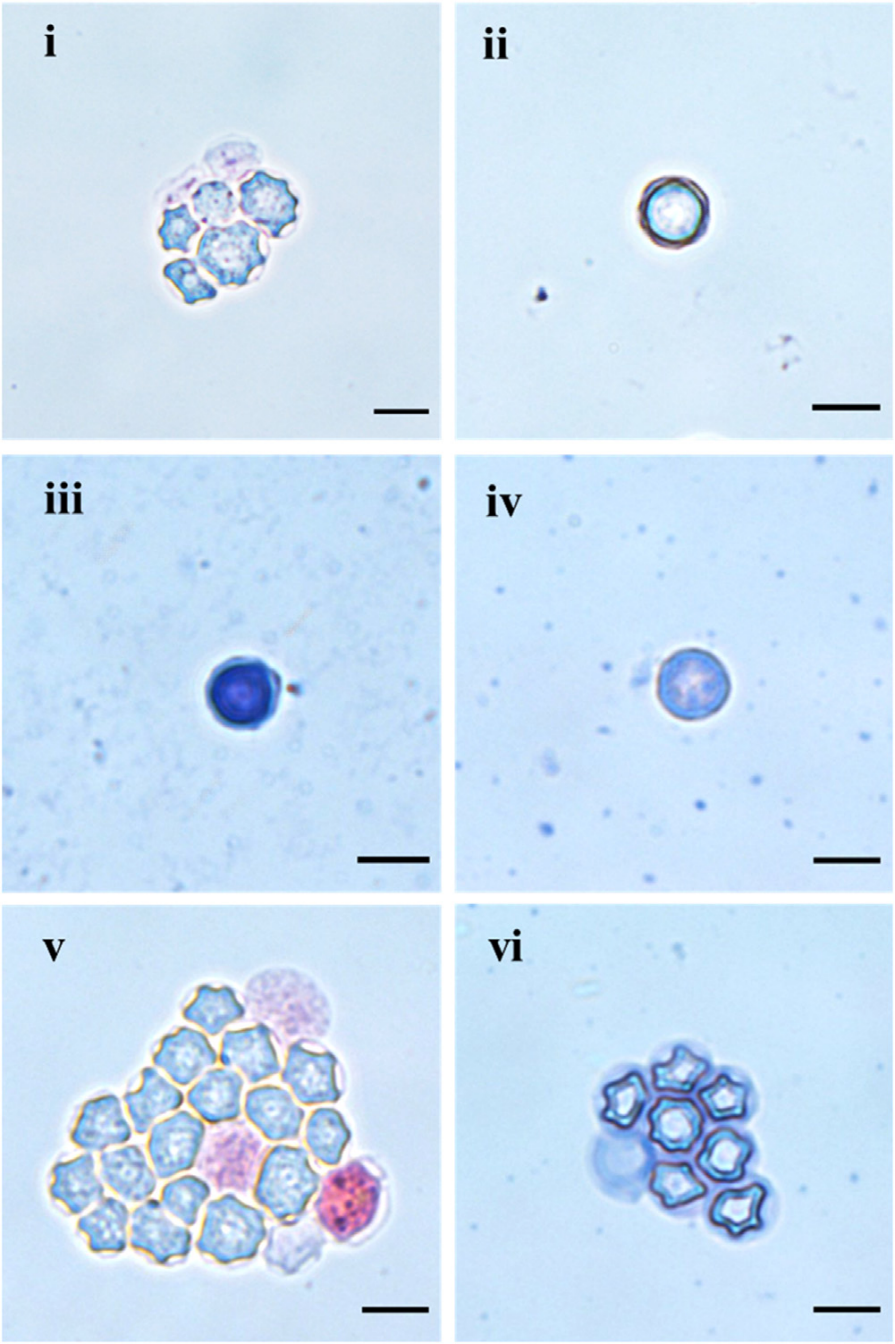
Representative morphological forms of *Acanthamoeba* cysts cultured from AK patients’ corneal swabs and their domestic tap water. (i) Cysts of isolate AK16-Cornea (*Acanthamoeba* sp. T4D) were with an average diameter of 16-22 μm. The ectocyst appears amorphous, and fine granular endocyst has 4-5 broad rays. (ii) Isolate AK15-Cornea cyst showing an average diameter of 12-16 μm, endocyst was almost spherical and ectocyst loosely followed the endocyst layer. (iii) Cyst of a water isolate (AK16-DW) *Acanthamoeba* sp., with an average diameter measuring 12-18 μm and a visible conical ostiole connecting the endocyst and ectocyst. The endocyst showed an almost rounded shape with subtle angles (iv). Cyst of a corneal isolate (AK12-Cornea) of *Acanthamoeba* sp. had an average diameter of 12-15 μm, with a spherical endocyst and thick ectocyst closely mimicked the contour of the endocyst. (v) Cysts of an *Acanthamoeba* sp. recovered from an AK patient (AK06-Cornea) had an average diameter of 11-16 μm, granular endocyst was irregularly polyhedral and folded ectocyst was loosely adhered to the endocyst. (vi) Mature cysts of an *Acanthamoeba* sp. isolated from domestic tap water (MK01-DW) measuring an average diameter of 11-15 μm. The endocyst mostly exhibited a pentagonal or quadrangular shape, while the ectocyst loosely encircled the endocyst, giving the cyst a round appearance outwardly. (i, ii, and v) stained with Eosin and (iii, iv, and vi) stained with Giemsa. Scale bar 10 μm. AK, *Acanthamoeba* keratitis.

The identification of *Acanthamoeba* trophozoites was facilitated by their large size (20–40 µm), along with the distinct and characteristic presence of acanthopodia resembling thorns, which protruded from the plasma membrane (Figure S4). In contrast, the double-walled cysts exhibited various shapes and sizes (11–22 μm), primarily characterized by a wrinkled and discontinuous ectocyst, along with uniform, thick, spherical, or polygonal endocysts displaying a granular surface texture (Figure 1i–vi). The cysts were mostly polygonal with varying numbers of arms (Figures 1i, 1v, and 1vi), suggesting that they belong to group II isolates according to the classification by Pussard and Pons [24].

**Figure 2:**
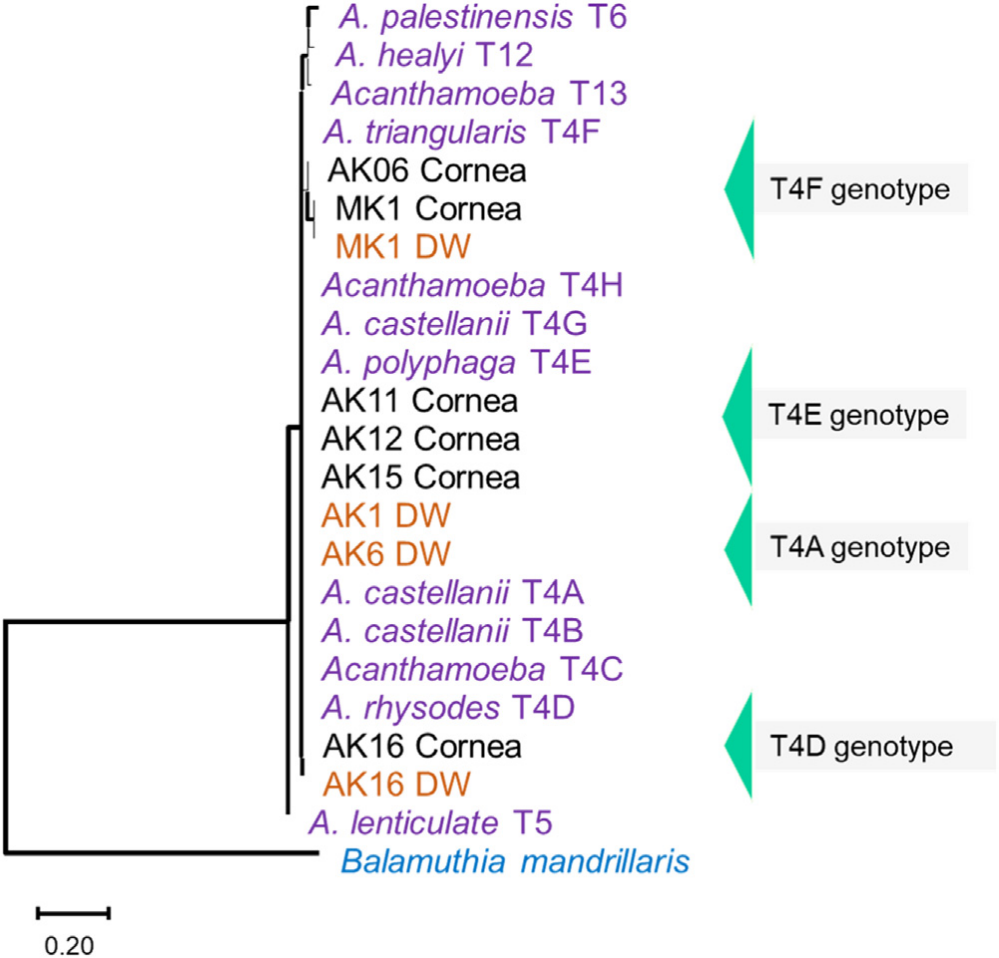
Phylogenetic tree based on the partial sequence of the 18S rRNA (*rns* gene) constructed using the neighbor-joining method with the Kimura-2 parameter algorithm and bootstrapping values calculated for 1000 replicates. All 10 *Acanthamoeba* isolates of the present study belonged to genotype T4, with four subclusters identified as T4A, T4D, T4FE, and T4F. Corneal isolates are labelled in black, while water isolates (‘DW’ represents domestic water) are labelled in orange. Reference species and genotypes, retrieved from the NCBI database are marked in purple. *Balamuthia mandrillaris* (KU184269, GenBank accession number) was used as an out group species.

Microscopically suspected *Acanthamoeba* isolates were confirmed through PCR (Figure S5) and sequencing. The *rns* gene sequence showing the highest alignment (% identity, ≥95%) with existing genotypes of *Acanthamoeba* in NCBI was assigned to each strain. All 10 *rns* gene sequences have been deposited in GenBank under accession numbers OR263296 to OR263305 (Table S1). Phylogenetic tree analysis based on the neighbor-joining method showed that all 10 *Acanthamoeba* isolates (six from corneal swabs and four from water samples) belonged to genotype T4. In the genotype T4 clade, four subclusters belonging to the T4A (two isolates), T4D (two isolates), T4E (three isolates), and T4F (three isolates)were observed (Figure 2). Among three cases showing *Acanthamoeba* culture-positive results for corneal and water samples, it was interesting to observe closely related corneal and water isolates in the phylogenetic tree for two patients (AK16 and MK1).

### Intracellular bacteria in Acanthamoeba isolates

**Figure 3:**
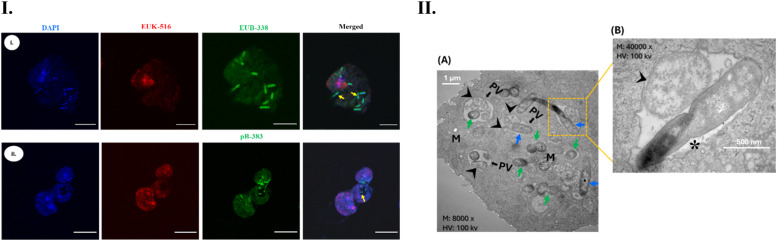
(I) Representative images of hybridization assay showing the presence of intracellular bacteria in *Acanthamoeba* trophozoites. Isolates AK06–cornea (i) and AK1–H_2_O (ii) were positive for intracellular bacteria. Probes EUK516 conjugated with Cy5 (red), targeting *Acanthamoeba*, and EUB338 conjugated with FITC (shown in green) targeting bacterial 16S rRNA were used. For AK1–H_2_O, probe pB–383 was used which specially binds with *P. aeruginosa* DNA. DAPI was used in mounting medium. Yellow arrow indicates bacterial cells undergoing binary fission. Scale bar 10 μm. 4.(II) TEM images showing ultrastructure of bacterial cells and intracellular niche within the *Acanthamoeba* host. Overview of the presence of intracellular bacteria within a corneal isolate of *Acanthamoeba* (A). At higher magnification, bacteria inside the phagocytic vacuole were observed, with rod-shaped bacteria undergoing binary fission (*), while cocci-shaped bacterial cell appeared disintegrated and digested within the PV (arrowhead) (B). Spherical and rod shaped bacteria were dispersed throughout the cytoplasm (A). Undigested cocci bacteria (appearing intact) and digested cocci bacteria were found within the same PV. Symbols: M: mitochondria; PV: phagocytic vacuole; green arrow: spherical bacteria; blue arrow: rod bacteria; asterisk (∗): binary fission; arrowhead: digested bacteria.

The presence of intracellular bacteria in axenically grown *Acanthamoeba* trophozoites was assessed by PCR, followed by FISH. Out of 10 *Acanthamoeba* isolates recovered in this cohort, only two strains (20%) harbored intracellular bacteria; one was a corneal strain (AK06-cornea) and the other was a water isolate (AK1-H_2_O) (Figure S6). The universal bacterial probe (EUB338) hybridized with trophozoites isolated from the cornea (AK06-cornea). *Acanthamoeba* sp. (AK1-H_2_O) cultured from domestic tap water harbored *P. aeruginosa*, as probe pB-383 (which specifically binds to *P. aeruginosa* 16S rRNA) hybridized with AK1-H_2_O trophozoites. In FISH, rod-shaped bacteria were seen within the trophozoites of both *Acanthamoeba* isolates (Figure 3.I). Under TEM, bacteria were observed throughout the cytoplasm, with some found within the phagocytic vacuole (PV) containing ingested bacteria. Both rod- and cocci-shaped bacteria were found within the same strain of *Acanthamoeba* (Figure 3.II).

### Demographics, clinical characteristics, and reported risk factors in AK patients

The mean age of the patients was 40.7 years (SD ± 12.3; 95% CI: 34.9 to 46.6 years) (Table S2). The proportion of AK in males (47.6%) and females (52.4%) was comparable. Furthermore, the proportion of culture-positive AK was not significantly (*P* = 0.06) higher in males (5/10) than in females (1/11) (Table S2). Two patients reported ocular trauma, while data for six patients were not available. Among the 21 AK patients, contact lens wear information was available for only 14 patients, and eight of them were contact lens wearers.

**Figure 4:**
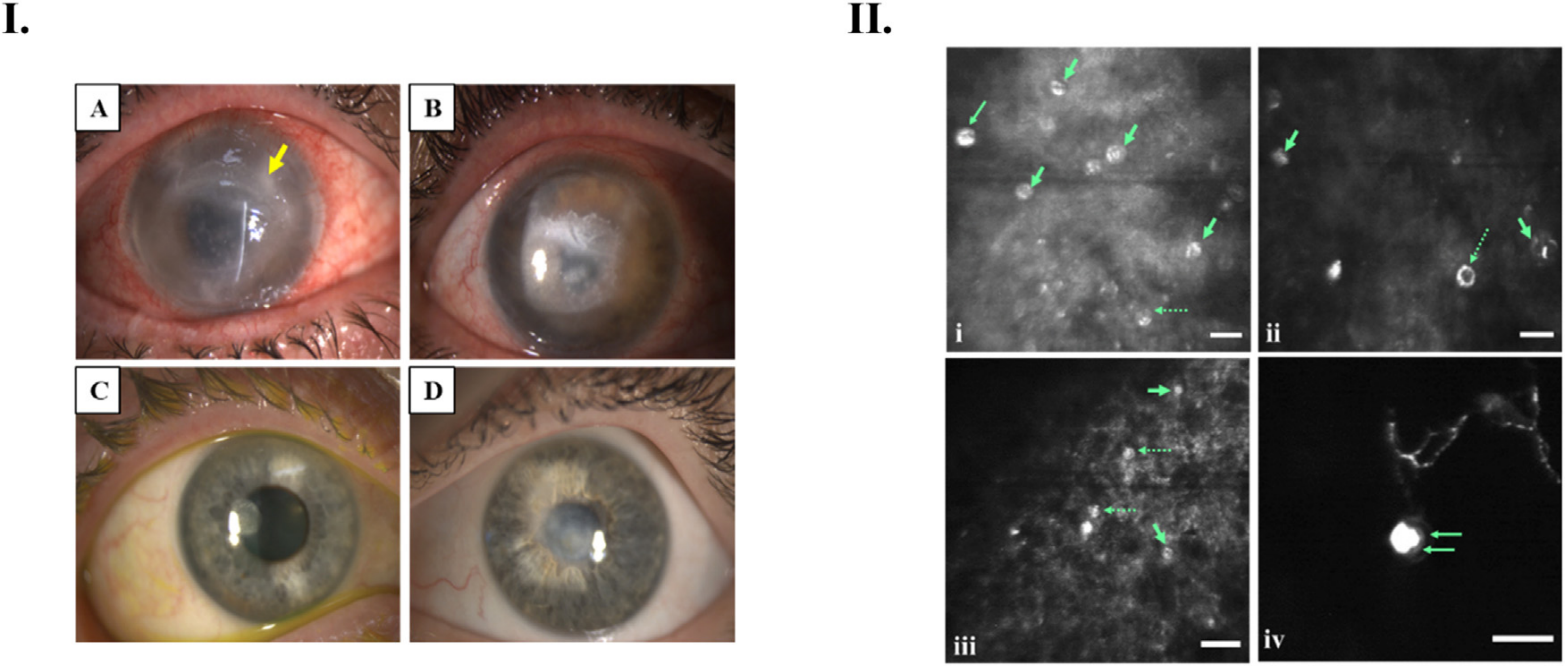
(I) Representative slit–lamp images of corneal ulcers infected with *Acanthamoeba* spp. in the eyes of AK patients during their first clinic visit after 2–3 weeks of infection. (A) Slit–lamp image showing diffuse corneal edema, superficial stromal ulcer, ditch–shaped melting at 6 o’clock position, slight neovascularization, incomplete ring infiltrate (yellow arrow) and multifocal stromal infiltrates. (B) AK patient’s cornea with stromal edema, superficial stromal ulcer, diffuse oedema, and ring abscess. (C) Early stage of AK showing subepithelial infiltrate and keratopathy. (D) Stromal edema, central subepithelial infiltrate, and roughness of central epithelium. 5.II: IVCM images showing cysts of *Acanthamoeba* in corneal surfaces of AK patients. The cyst displays a bright centre (nucleus), encircled by a ring–like wall with low refractivity (i-iii). The central structure is spherical and exhibits a uniform level of reflection (i). In some cases, the cyst wall appears hyperreflective with a dark central area resembling a hollow ring (indicated by dashed arrows, [i-iii]). A double-walled cyst (long arrows) with a polygonal endo–cyst wall (iv). Scale bar 20 µm.

Twelve patients (57.1%) responded to the risk factors survey, and among them, six were contact lens wearers. Five were soft contact lens users, while one was a rigid gas permeable (RGP) lens wearer. Eleven patients reported experiencing the first symptom of AK in indoor environments, either in their residence or office spaces, while one patient noticed it in an industrial area. One of the patients flushed their eyes with water and saline due to severe pain, and another had a history of sleeping while wearing contact lenses. Five wearers used their domestic bathroom spaces for lens insertion, two had a history of swimming while wearing contact lenses, and one showered with lenses. Among six wearers who responded to the survey questionnaire, five (83.3%) believed that AK infection might be associated with contact lens wear. Eye pain was the most common presenting symptom in 14 cases (66.7%), followed by red eye in 11 cases. Clinically, most of the ulcers had single or multiple cysts around the lesions. Two patients had ring infiltrates, and neovascularization was observed in one patient (Figure 4.I). Under IVCM, *Acanthamoeba* cysts were characterized by a highly reflective central nucleus surrounded by a less refractive cyst wall (Figure 4.II). Cysts had diameters of 8–22 μm with a regular spherical shape and uniform reflection. A few cysts with irregular shapes, such as hollow/signet ring, were also observed (Figure 4.II).

The primary therapy for the ulcer consisted of polyhexamethylene biguanide (PHMB) in 17 cases and chlorhexidine in two cases, with 16 cases additionally receiving broad–spectrum antibiotics such as chloramphenicol, doxycycline, moxifloxacin, ofloxacin, ciprofloxacin, fortified cefazoline, and gentamycin. Three cases also received antiviral drugs (acyclovir and valaciclovir), and two cases received the antifungal voriconazole. A patient who had a recurrent ulcer and another patient who needed a corneal graft had prolonged treatments of 78 and 64 weeks, respectively (Table 1). The median duration of treatment was 12 weeks (8–26 weeks, interquartile range).

**Table 1:** Major clinical characteristics of patients with *Acanthamoeba* keratitis.

S.N.	Patient code	Age (years), gender	Symptoms at presentation	Symptoms duration at presentation (days)	Ulcer			Hypopyon	VA in affected eyes		Main treatment	Treatment duration (weeks)	*Acanthamoeba* culture and PCR	Final status
					Location	Depth	Size (mm)		Presenting	Final				
1	AK00	57, F	Eye pain, decreased vision	>180	Central and paracentral	Deep	6.1×5.8	No	3/60	6/18 (6/9 with PH)	PHMB, dexamethasone, moxifloxacin, and oral prednisolone	52	Negative	Healed with calcific scars
2	AK01	45, F	Eye pain, red eye, photophobia	7	Central	Superficial	1.8×1.8	No	6/18	6/7.5	Dexamethasone, chlorhexidine, and ofloxacin	26	Negative	Healed with streak–like corneal scars
3	AK02	34, F	Epiphora, FBS, photophobia	NA	Central	Superficial	1.7×1.7	No	6/24	6/6	PHMB, ofloxacin, and propamidine isethionate	44	Negative	Healed with scar
4	AK03	40, M	Eye pain, red eye	NA	Paracentral	Superficial	NA	No	NA	NA	PHMB, and propamidine isethionate	8	Negative	Healed
5	AK04	37, M	Eye pain	20	Paracentral	Superficial	NA	No	6/30	6/9	PHMB, ofloxacin, and propamidine isethionate	8	Negative	Healed
6	AK06	51, M	Eye pain	30	Central	Superficial	2×2	NA	CF at 2 feet	6/30	PHMB, ofloxacin, acyclovir, ofloxacin, and chloramphenicol	12	Positive	Healed with an apical scar
7	AK07	39, F	Eye pain, discharge, swelling, red eye	10	Paracentral	Superficial	2 x 3	No	6/24	6/12	Oral doxycycline, ofloxacin, propamidine isethionate	8	Negative	Healed with a fainty patchy scar
8	AK08	58, F	Eye pain, decreased vision, photophobia, red eye	>360	Central and paracentral	Deep	NA	NA	PL+	NA	Chlorhexidine, ofloxacin, ciprofloxacin, and voriconazole	64	Negative	Corneal graft
9	AK09	60, F	NA	7	Central	Superficial	1.7×2.6	No	4/60	6/24	PHMB, ofloxacin, and chloramphenicol	4	Negative	Healed with scars
10	AK10	20, F	Eye pain, discharge	110	Paracentral	Superficial	0.8×0.8	No	NA	6/7.5	PHMB, ofloxacin, and chloramphenicol	12	Negative	Healed with a scar
11	AK11	40, M	Blurred vision, eye pain, discharge	30	Central	Superficial	1×1	No	NA	6/18 (6/9 with PH)	PHMB, and ofloxacin	12	Positive	Healed with a scar
12	AK12	19, M	Red eye, eye pain	7	Central	Deep	NA	No	NA	6/24	PHMB, and ofloxacin	8	Positive	Healed
13	AK13	37, F	Photophobia, red eye	360	Central and paracentral	Deep	3.5×2.8	No	2/60	6/18	Chloramphenicol, ofloxacin, prednisolone, PHMB, valacyclovir, and voriconazole	78	Negative	Recurrent
14	AK14	32, M	Red eye, eye pain	14	Peripheral	Superficial	NA	No	6/12	6/9	PHMB, ofloxacin, chloramphenicol, and valacyclovir	12	Negative	Healed
15	AK15	39, F	NA	21	Central	Deep	2.5×2.5	No	NA	6/12	PHMB	20	Positive	Healed with scar
16	AK16	39, F	Eye pain, red eye	14	Paracentral	Superficial	NA	NA	NA	6/7.5	NA	4	Negative	NA
17	AK21	50, M	Red eye	NA	Central	Deep	NA	NA	NA	NA	PHMB, and ofloxacin	NA	Positive	NA
18	MK01	28, M	Red eye, pain	90	Central and paracentral	Deep	0.7×0.6	No	3/60	6/12	PHMB, and chloramphenicol	20	Positive	NA
19	MK02	44, M	NA	28	Central	Superficial	NA	NA	NA	6/6	PHMB, and chloramphenicol	NA	Negative	NA
20	MK05	63, M	Photophobia, red eye	21	Central and paracentral	NA	2/3^rd^ of cornea	Yes	2/60	NA	PHMB, and ofloxacin	12	Negative	NA
21	MK03	23, F	Photophobia, eye pain	28	Diffused and Paracentral	Superficial	NA	No	2/60	6/48	PHMB, and ofloxacin	8	Negative	NA

DM, descemet membrane; F, female; FBS, foreign body sensation; KP, keratic precipitate; M, Male; NA, not available; PH, pinhole; PL, perception of light; PHMB, polyhexamethylene biguanide; RGP, rigid gas permeable; VA, Visual acuity.

Out of the 21 corneal swabs (collected along with ulcer debridement) cultured from AK patients, only six (28.5%) tested culture-positive for *Acanthamoeba* spp. (Table 1). Among the 15 culture-negative cases, six (40%) had been started on either PHMB or chlorhexidine treatment before their referral to the hospital. The mean symptom duration at presentation was 35.6 ± 31.8 days among culture-positive cases, compared to 115.4 ± 130.2 days among culture-negative cases, but the difference was not significant (*P* = 0.8). Surprisingly, the mean treatment duration of culture-negative cases was higher (25.5 ± 24.7 weeks) compared to positive cases (14.4 ± 5.4 weeks), although this was not significant (*P* = 0.86). *Acanthamoeba* was cultured from both patients who reported ocular trauma. One of the culture-negative AK patients (AK13) was treated with a topical corticosteroid in conjunction with PHMB, antibiotics, antiviral agents, and voriconazole, but the ulcer did not resolve even after 78 weeks of treatment. Two other culture–negative AK patients, AK00 and AK01, were also treated with a topical corticosteroid along with PHMB and chlorhexidine, respectively. Their ulcers healed, leaving calcific scars.

## Discussion

This is the first cross-sectional study in Australia on AK with active participation of patients to collect their domestic tap water samples and gain insight into patients’ perspectives on potential risk factors associated with AK. During the 1.25-year study at a referral Eye Hospital in Sydney, 21 AK patients had their corneal swabs cultured along with ulcer debridement, and all had their demographic and clinical characteristics reviewed. Additionally, domestic tap water samples were analyzed from 42.8% of the AK patients enrolled in the study, and 57.1% of the patients responded to the risk factors questionnaire survey. Six *Acanthamoeba* isolates recovered from corneal samples and four from domestic tap water were genotyped and assessed for the presence of intracellular bacteria. Most of the AK cases presented in summer and spring, consistent with previous reports of higher cases during warmer months [25]. This is likely due to *Acanthamoeba*’s preference for warmer habitats and increased water activities of people during the summer [26].

Previous retrospective audits conducted in different states of Australia have identified wearing contact lenses coupled with water exposure as a significant risk factor for AK [[14], [15], [16]]. This study made a similar observation, where among the individuals with information available about contact lens use, 57.1% (8/14) of AK patients were lens wearers, and 62.5% (5/8) of them wore soft lenses. Among lens wearers, 37.5% had a history of engaging in water activities such as swimming or showering while wearing their lenses. It is well established that contact with contaminated water is a leading risk factor for AK, and the current study found that a higher proportion of AK patients had a domestic water supply contaminated by *Acanthamoeba* spp. Isolation of *Acanthamoeba* spp. from both corneal and domestic water samples of three AK patients of this cohort, with two of the same genotype group, further supports the advice to not use tap water in contact lens care routines.

While *Acanthamoeba* culture has a relatively low sensitivity and positivity rate, it remains the current gold standard for diagnosing AK [11,12]. However, when PCR–based assays are combined with culture and IVCM, sensitivity usually increases significantly [13]. In the current cohort, 40% (6/15, excluding six cases who had started PHMB before the collection of culture specimens) of corneal specimens grew *Acanthamoeba* spp., and notably, culture-positive cases had a symptom duration at presentation over three times shorter than culture-negative cases. Nearly 43% of culture-negative cases had started antiamoebic therapy (AAT), while the remaining cases had begun treatment with either antibiotics, antivirals, antifungals, or a combination of these drugs before collecting corneal specimens for culture. Initiation of AAT could decrease the culture’s sensitivity, as topical medication can clear superficial ulcers but not those that have deeper infections [27].

Current data on the circulating genotypes of *Acanthamoeba* strains isolated from AK patients in Australia are limited, with only one case of genotype T5 reported from South Australia [28], while other isolates have been classified based on unreliable cyst morphology in the literature [29], which is often influenced by *in vitro* growth conditions [30]. The predominant genotype found in domestic tap water [18,20] and public lagoons [17] in the greater Sydney area was T4. Additionally, genotypes T2 [17], T3 [20], and T5 [17,18] were also identified in water samples. Genotypes T3 [31] and T5 [32] are occasionally reported from AK patients. As reported globally [21], all six corneal isolates and four water isolates in the current study belonged to genotype T4, and four DF3 variants were identified within the T4 genotype. Furthermore, two water isolates were closely related to the corresponding corneal isolates in the phylogenetic tree, indicating tap water as a potential source of infection; however, high–throughput whole genome sequencing is required to confirm their origin from the same source.

Among 10 *Acanthamoeba* isolates of this study, one corneal and one water isolate tested positive for intracellular bacteria. The patient, who was infected by *Acanthamoeba* sp. with intracellular bacteria, visited the hospital a month after the initial symptoms manifested, presenting with sloughy corneal epithelium and a significant impairment in visual acuity. The case was treated with PHMB, antibiotics, and antiviral drugs. After 12 weeks, the patient's vision improved, but an apical scar in the cornea was reported. Similarly, a previous study has shown that AK patients infected by *Acanthamoeba* strains with intracellular bacteria exhibited enhanced epithelial defects and a higher proportion of stromal infiltrates [21]. Another study has observed worse initial VA, prolonged time to diagnosis, and extended symptom duration at presentation in the presence of intracellular bacteria [33]. The release of intracellular bacteria in a compromised cornea could enhance inflammation and potentially exacerbate clinical outcomes [33].

## Conclusion

This report is the first to assess the circulating genotypes of *Acanthamoeba* spp. among AK patients in the Sydney metropolitan area, and it identified T4 as the prevalent genotype in both corneal specimens and patients' domestic tap water samples. The current study cohort suggests that *Acanthamoeba* ocular infection is still rare in Sydney but may have a higher annual incidence of AK than reported in the literature. Thus, eye practitioners in the greater Sydney area should always suspect *Acanthamoeba* as a potential cause of contact lens-related keratitis, irrespective of the season. The study also provides data confirming that contact lens wear coupled with water exposure is the primary risk factor for AK in the study cohort.

## Declarations of competing interest

The authors have no competing interests to declare.

## Appendix Supplementary materials

Image, application 1
