# Supplementary material for: Clinical presentation, genotypic diversity, and intracellular bacteria in Acanthamoeba keratitis patients treated at a referral eye hospital in Sydney, Australia
Source: IJID Reg. 2025 Jun 15;16:100690. doi: 10.1016/j.ijregi.2025.100690 (PMC12271423; doi:10.1016/j.ijregi.2025.100690)
Supplement: Supplementary file 1 [file mmc1.pdf]

Table S1: Oligonucleotide probes used in this study for hybridization assay

S.N.	Probe name	Sequence (5′ → 3′)	rRNA position	Conjugated with	Specificity	Refs.
1.	EUK516	ACCAGACTTGCCCTCC	502–517 (18S)	Cy5	Eukaryota	(1)
2.	EUB338	GCTGCCTCCCGTAGGAGT	338–355 (16S)	FITC	Most eubacteria	(2)
3.	pB-383	GGTAACCGTCCCCCTTGC	1449-1466 (16S)		*P. aeruginosa*	(3)

STUDY FLOW CHART - 1

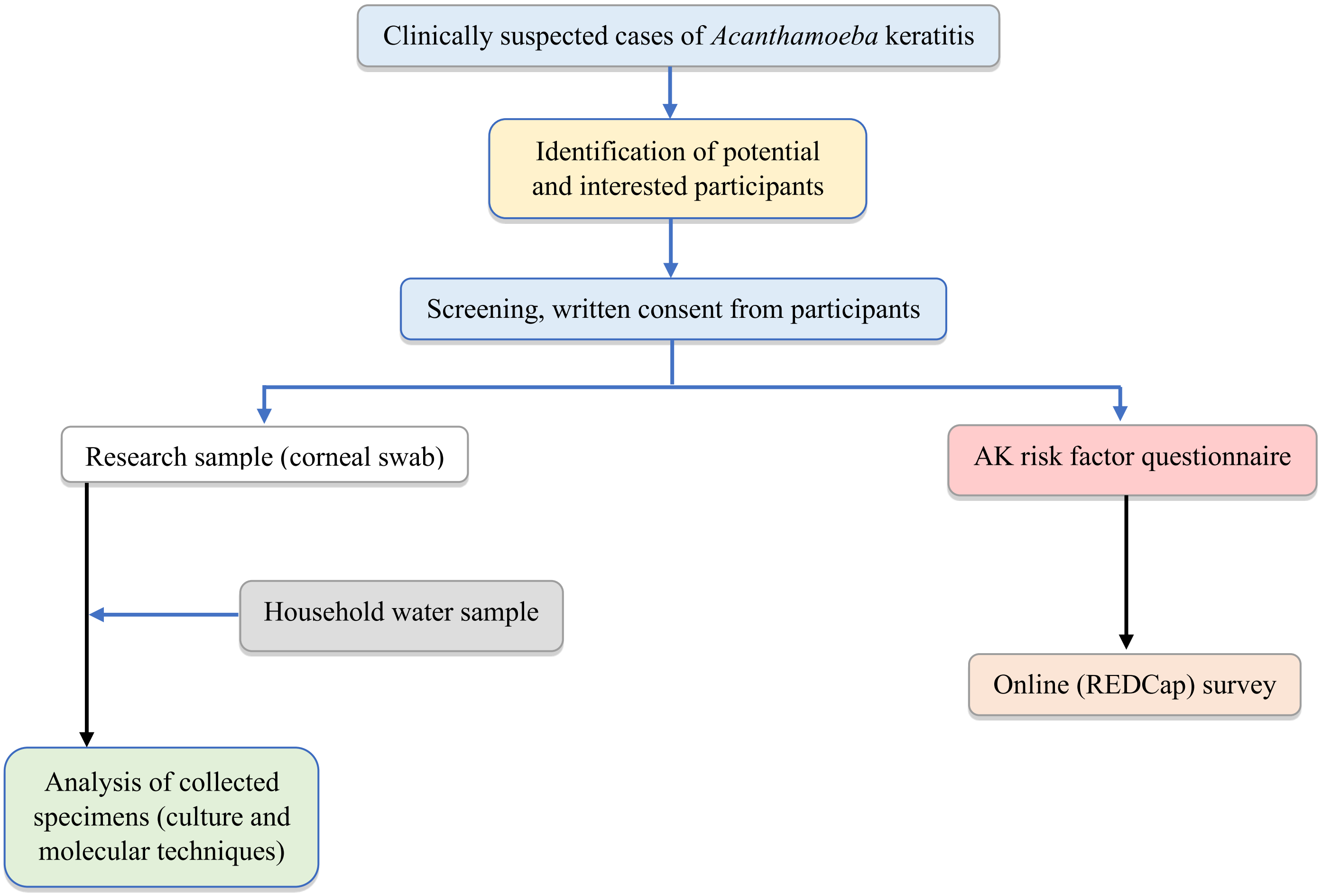

Appendix A

Water Sample Collection Instructions

EQUIPMENTS
You are provided the following equipment in a zip-lock bag:

A. Sterile polyester tipped applicator.

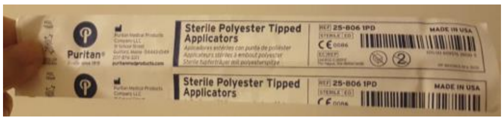

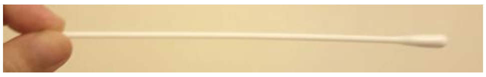

B. 15 ml sterile test tube with an orange cap.

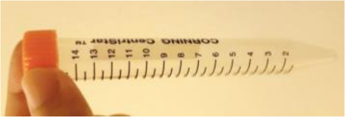

GENERAL INSTRUCTIONS:
1. The steps on should be performed in the morning BEFORE the bathroom is used.
2. Following collection of samples, place the tube back in the zip-lock bag, complete the questionnaire provided, and return the sample and questionnaire to the researchers as soon as possible.
3. Samples should be stored between 0-46 °C, therefore we recommend that samples are NOT stored in the fridge or freezer and are NOT left in the car.

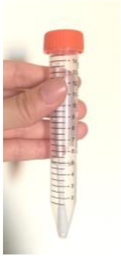

STEP 1
Unscrew the orange cap of the tube.

STEP 2
Open the packet containing the sterile polyester tipped applicator.

STEP 3
Wipe the swab around the inside of the bathroom sink drain for 10 seconds then put swab inside the tube and brake off the shaft of swab, so that swab fits into the tube (as shown on the right).

STEP 4
Turn on the COLD tap <u>slowly</u> and add a small amount of water into the tube until it approximately reaches the line next to the '5'.

STEP 5
Place the swab in the tube and screw the orange cap tightly to ensure that the water does not leak out.

Questionnaire

Sample collection date	/ /
Sample collection time	: am/pm
Suburb	
Postcode	

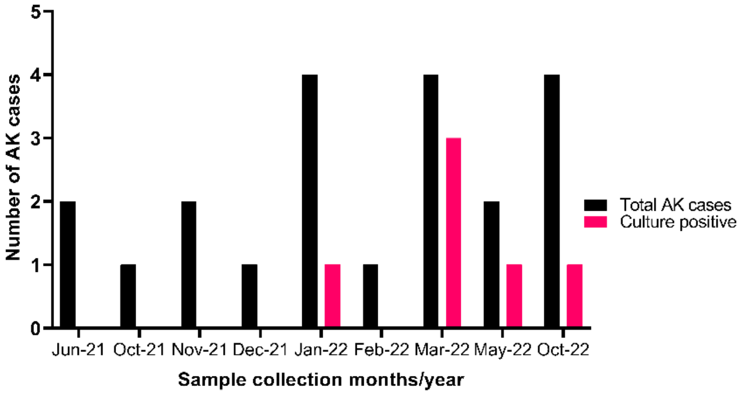

Fig. S1: The total number of *Acanthamoeba* keratitis (AK) cases and culture confirmed cases per month and year of the study period. Months with no cases were removed from the graph to improve visualisation.

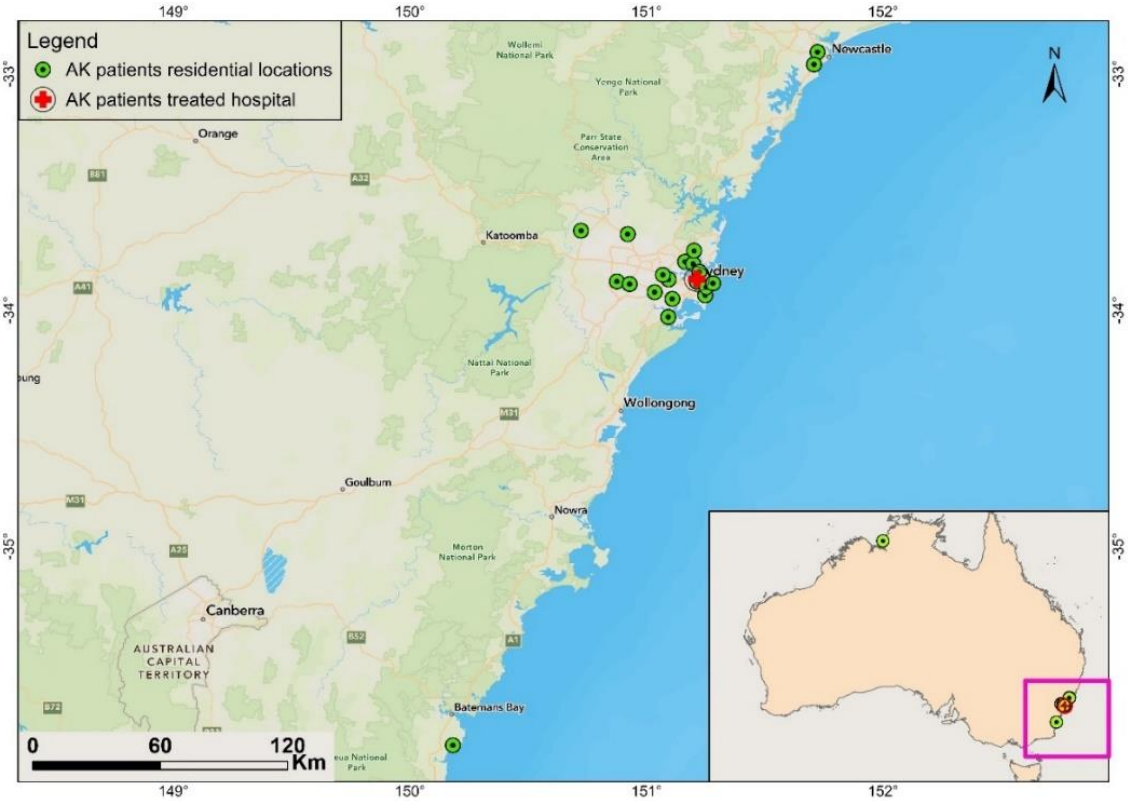

Fig. S2: Map showing the residential location of *Acanthamoeba* keratitis (AK) patients enrolled in this study. Among the 21 AK patients enrolled, 20 (95.2%) were from different suburbs of the greater Sydney region (shown in enlarged map section), except for one case from the Northern Territory of Australia. The map was created using ArcGIS (Esri GIS, California, USA).

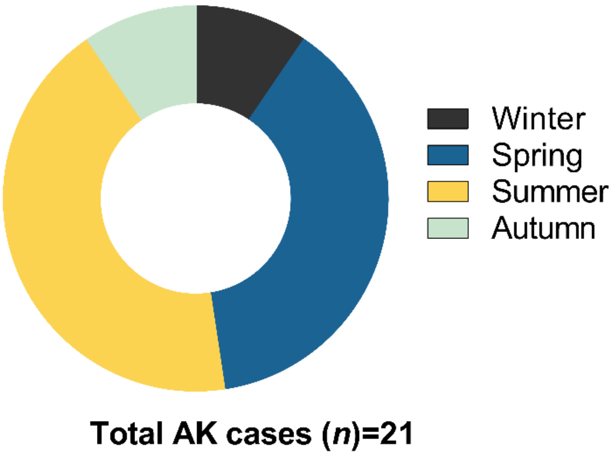

Fig. S3: Number of AK patients recruited during the study period from June 2021 to October 2022 at Sydney Eye Hospital, Sydney, Australia. Corneal swabs along with ulcer debridement specimens were collected for the culture of *Acanthamoeba* spp. from each patient.

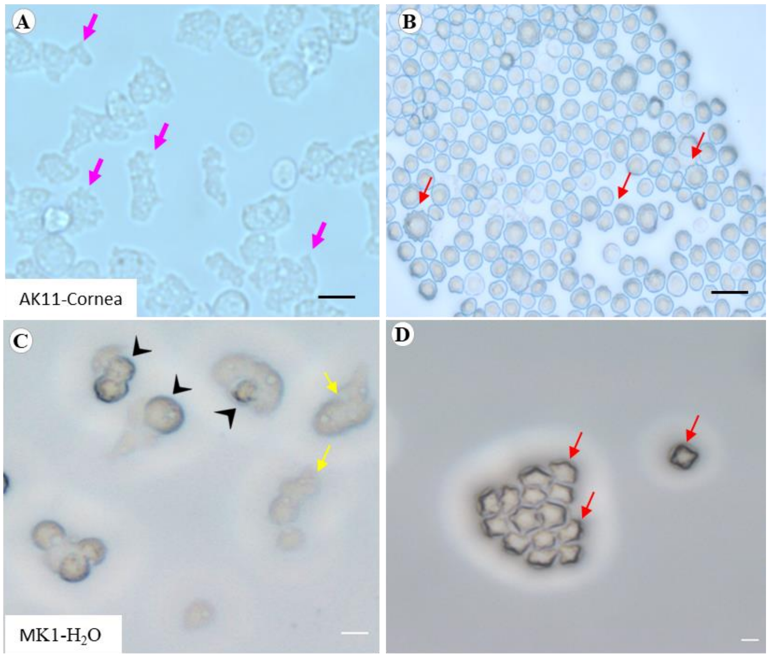

Fig. S4: Representative microscopic images of *Acanthamoeba* trophozoites and cysts. Trophozoites (**A**) in PYG medium supplemented with penicillin-streptomycin and mature cysts on NNA plate (**B**). This strain was recovered from corneal swab (AK11) of a patient with AK (**A-B**) and trophozoites are with visible thorn like acanthopodia (pink arrows) (**A**). Some trophozoites (yellow arrow) and a few cysts in the encystment stage (arrowhead) can be observed (**C**), along with mature cysts both in groups and individually on NNA plate (**D**). This strain was isolated from domestic tap water of a patient with AK (MK1-H_2O) (**C-D**). Indicators: Pink arrow, acanthopodia; yellow arrow, trophozoites; red arrow, irregular polygonal cysts; and arrowhead, emergence stage of encystment. Scale bar 10 µm.

A. **B.**

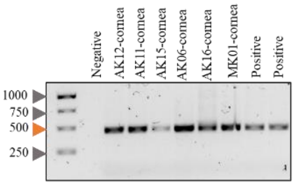

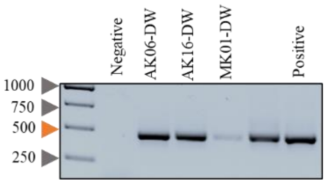

Fig. S5: The agarose gel images in (**A**) show PCR amplicons of *Acanthamoeba* strains isolated from corneal specimens, while (**B**) depicts samples from domestic tap water of AK patients. The bands, visualized using 2% gel electrophoresis, represent the ~450 bp amplicon produced by the JDP1/2 primer. *A. culbertsoni* (ATCC 30171) was used for positive control, and Milli–Q water for negative reactions. "DW" indicates domestic water.

Table S1: Identification of *Rns* genotypes of *Acanthamoeba* strains isolated from corneal specimens (*n*=6) and domestic tap water (*n*=4) of AK patients

S.N.	Strain ID	Sample source	NCBI BLASTn result for *Rns* genotype	GenBank accession no	Sequence identity of isolates to published strains (% identity, highest homology), accession number
1.	AK12	Cornea	*Acanthamoeba* T4	OR263296	*Acanthamoeba* sp. T4 (100%, 384/384 bp), MG386312
2.	AK11		*Acanthamoeba* T4	OR263297	*Acanthamoeba* sp. T4 (99%, 403/404 bp), JX423592
3.	AK15		*Acanthamoeba* T4	OR263298	*Acanthamoeba* sp. T4 (98%, 379/384 bp), MN700296
4.	AK06		*Acanthamoeba* T4	OR263299	*Acanthamoeba* sp. T4 (98%, 411/420 bp), KU936117
5.	AK16		*Acanthamoeba* T4	OR263300	*Acanthamoeba* sp. T4 (100%, 387/387 bp), MH620481
6.	MK01		*Acanthamoeba* T4	OR263301	*Acanthamoeba* sp. T4 (95%, 388/407 bp), MH790986
7.	AK1	Water	*Acanthamoeba* T4	OR263302	*Acanthamoeba* sp. T4 (98%, 348/354 bp), KU936111
8.	AK06		*Acanthamoeba* T4	OR263303	*Acanthamoeba* sp. T4 (99%, 398/403 bp), KJ094685
9.	AK16		*Acanthamoeba* T4	OR263304	*Acanthamoeba* sp. T4 (100%, 330/330 bp), MH620481
10.	MK01		*Acanthamoeba* T4	OR263305	*Acanthamoeba* sp. T4 (95%, 385/405 bp), KT985972

i. **ii.**

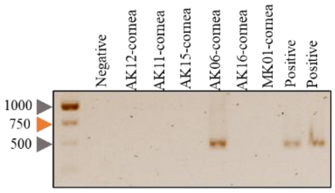

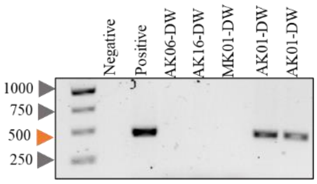

Fig. S6: Axenically grown *Acanthamoeba* isolates in PYG broth were gauged for the presence of intracellular bacteria using 16S rRNA primer 341fw/785rv (V3–4) (~464 bp). *E. coli* (ATCC 10798) was used for 16S rRNA positive control, and Milli–Q water for the negative.

Table S2: Descriptive demographic data of AK patients recruited in this cohort (*n*=21)

**Demographic**	***Acanthamoeba* culture positive (*n*=6)**	***Acanthamoeba* culture negative* (*n*=15)**
**Patients number** (*n*)	6	15
**Age (years)**		
Mean±SD (range)	37.8±12.5 (19–51)	41.9±12.9 (20–63)
**Gender**		
Male [n(%)]	5 (83.3)	5 (33.3)
Female [n(%)]	1 (16.7)	10 (66.7)
**Contact lens wear (current)** [n(%)]		
Contact lens use	3 (50)	5 (33.3)
Missing data (*n*)	2	4
**Ocular trauma** [n(%)]	2 (33.3)	0
Missing data (*n*)	2	4

*Culture negative cases of AK were confirmed through IVCM and slit–lamp observation.

References

1. Amann RI, Binder BJ, Olson RJ, Chisholm SW, Devereux R, Stahl DA. 1990. Combination of 16S rRNA-targeted oligonucleotide probes with flow cytometry for analyzing mixed microbial populations. Appl Environ Microbiol 56:1919-25.
2. Amann RI, Krumholz L, Stahl DA. 1990. Fluorescent-oligonucleotide probing of whole cells for determinative, phylogenetic, and environmental studies in microbiology. J Bacteriol 172:762-70.
3. Hogardt M, Trebesius K, Geiger AM, Hornef M, Rosenecker J, Heesemann J. 2000. Specific and rapid detection by fluorescent in situ hybridization of bacteria in clinical samples obtained from cystic fibrosis patients. J Clin Microbiol 38:818-25.
